# LncRNA SNHG15 regulates osteosarcoma progression *in vitro* and *in vivo* via sponging miR-346 and regulating TRAF4 expression

**DOI:** 10.1515/biol-2020-0039

**Published:** 2020-07-03

**Authors:** Xuewu Chen, Hongguang Xu

**Affiliations:** Department of Spine Surgery, Research Center of Spine Surgery, Yijishan Hospital (The First Affiliated Hospital of Wannan Medical College), No. 2, West Zheshan Road, Wuhu, 241001, Anhui, China

**Keywords:** osteosarcoma, SNHG15, miR-346, TRAF4

## Abstract

Osteosarcoma (OS) is a common primary malignant bone tumor around the world. It has been reported that long noncoding RNAs (lncRNAs) take part in diverse pathological processes of OS; however, the mechanism remains unknown. This study aimed to uncover the profile of lncRNA *small nucleolar RNA host gene 15* (SNHG15), its biological function, and its potential involvement in the mechanism of OS progression *in vitro* and *in vivo*. The expression of SNHG15 and TRAF4 was promoted in OS tissues opposite for that of miR-346. The silencing of SNHG15 limited the proliferation, invasion, and enhanced apoptosis of SaoS2 and HOS cells. Moreover, the putative binding sites between miR-346 and SNHG15 or TRAF4 were predicted by starBase and Targetscan software online, individually. Also, miR-346 deletion reversed the positive effects of SNHG15 elimination on proliferation, apoptosis, and invasion in cells. In addition, the upregulation of TRAF4 disrupted the biofunctional results from miR-346 promotion subsequently. Finally, SNHG15 knockdown repressed OS tumor growth in a xenograft tumor model. SNHG15 enhanced the progression of OS by regulating the miR-346/TRAF4 axis *in vitro* and *in vivo*.

## 1 Introduction

Osteosarcoma (OS) is the most common primary malignant bone tumor in children and adolescents [1], which was characterized by strong invasiveness and early metastasis [2]. Although chemotherapy regimens and neoadjuvant techniques have improved, approximately 50% of all patients have metastases at the time of diagnosis, which results in a high mortality rate [3]. Therefore, exploring the mechanism of OS is of great significance for understanding the progress of the disease and for finding potential therapeutic targets.

Long noncoding RNAs (lncRNAs) are a category of long RNAs (more than 200 nucleotides (nts) in length) without translation capacity that can affect gene expression at the transcriptional stage [4]. Emerging evidence suggests that lncRNAs act as functional regulators in tumorigenesis of cancers such as papillary thyroid carcinoma [5], gastric cancer [6], colorectal cancer [7], hepatocellular carcinoma [8], and pancreatic adenocarcinoma [9]. Extensive research indicates that the disordered expression of lncRNA is found in OS [10,11]. *Small nucleolar RNA host gene 15* (SNHG15), located on chromosome 7p13, is reported to be involved in the response to environmental stressors [12,13]. Recently, increasing evidence suggests that lncRNA SNHG15 is highly expressed in OS tissues and cells and regulates a range of processes, including proliferation, invasion, migration, and even autophagy [14], but the role of SNHG15 in OS has not been investigated in depth.

Numerous studies have shown that lncRNA can act as a molecular sponge in combination with miRNA to regulate the development of cancer [15,16,17]. It was reported that SNHG15 could mediate the development of cancer by binding a variety of miRNAs [14,18,19]. However, the targeting relationship between SNHG15 and miRNA is still ambiguous in OA. Previous studies have shown that miR-346 plays a role in tumor supõpression in OS [20]. In this experiment, we used starBase software to predict the possible binding of SNHG15 to miR-346.

Tumor necrosis factor receptor-associated factors (TRAFs) were originally discovered as adaptor proteins that regulate the cell life and death [21]. Tumor necrosis factor receptor-associated factor 4 (TRAF4) is a unique member of the TRAF protein family [22]. A recent article showed that TRAF4, working as an oncogene, was highly expressed in OS [23]. In the current study, we used Targetscan software to predict that miR-346 may target TRAF4. However, the molecular mechanisms of SNHG15, miR-346, and TRAF4 in OS remained unclear.

In this study, we first investigated the expression level of SNHG15 on OS tissues and cells and examined the changes in the biological phenotype of OS cells by loss-function of SNHG15. To explain the mechanism, we proposed that SNHG15 functions as a sponge of miR-346, allowing enhanced TRAF4 expression, thereby boosting the development of OS. Our findings provided a certain basic value for clinical diagnosis.

## 2 Materials and methods

### 2.1 Clinical samples

Thirty pairs of specimens from OS patients and healthy volunteers were collected from Yijishan Hospital (The First Affiliated Hospital of Wannan Medical College). All samples were preserved at −80°C. The main inclusion criteria were as follows: (1) patients were pathologically diagnosed with OS and (2) OS was the primary tumor, without chemotherapy, surgery, or radiotherapy. The exclusion criteria were as follows: (1) history of other malignant disease, (2) recurrent or previously treated disease, and (3) patients who received treatment within the past 90 days before admission.


**Informed consent:** Informed consent has been obtained from all individuals included in this study.
**Ethical approval:** The research related to human use has been complied with all the relevant national regulations, institutional policies, and in accordance with the tenets of the Helsinki Declaration and has been approved by the Ethics Committee of Yijishan Hospital (The First Affiliated Hospital of Wannan Medical College).

### 2.2 Cell culture and transfection

Human OS cell lines (MG63, U2OS, SaoS2, and HOS) and human osteoblast cell lines (HFOB1.19) were obtained from Shanghai Innovation Biotechnology Co., Ltd (Shanghai, China) and were cultured with 1% penicillin/streptomycin (Beyotime Biotechnology Company, Shanghai, China) as previously described [24]. Small interference RNA (siRNA) targeting SNHG15 (si-SNHG15), SNHG15 overexpression plasmid (SNHG15), TRAF4 overexpression plasmid (TRAF4), miR-346 mimic (miR-346), miR-346 inhibitor (miR-346 inhibitor), and controls (si-control, vector, miR-control, inhibitor-control) were all obtained from GenePharm (Shanghai, China). Lipofectamine 3000 (Invitrogen, Carlsbad, CA, USA) kit was used for transfection according to the manufacturer’s instructions. The sequences were shown as follows: si-SNHG15 (Sense: 5′-ACGGTGGCAACGTGCGTGGCCA-3′, Anti-sense: 3′-GCCTGCAACGGTGCAAAT GCG-5′).

### 2.3 Quantitative real-time polymerase chain reaction (qRT-PCR)

Total RNA of cells was extracted through TRIzol reagent (Life Technologies Corporation, Carlsbad, CA, USA). Primer-Script one-step RT-PCR kit (Takara, Shiga, Japan) or miRNA Reverse Transcription kit (GeneCopoeia, FulenGen, China) was employed to synthesize the first-strand complementary DNA of miR-346 and TRAF4. The levels of SNHG15, miR-346, and TRAF4 were assessed via SYBR Premix Dimer Eraser Kit (Takara). The primer sequences used are as follows: SNHG15 forward (5′-CAACCATAGCGGTGCAACTGTGC-3′), SNHG15 reverse (5′-GGCTGAACCAAGTTGCAAGTCATG-3′), miR-346 forward (5′-CACGGATCCCTTGTCAGCAAGGAGTG-3′), miR-346 reverse (5′-CGGAATTCTAGGTTGGGAGCGAAGTG-3′), TRAF4 forward (5′-AGGAGTTCGTCTTTGACACCATC-3′), TRAF4 reverse (5′-CTTTGAATGGGCAGAGCACC-3′), U6 forward (5′-GCTTCGGCAGCACATATACTAAAAT-3′), U6 reverse (5′-CGCTTCACGAATTTGCGTGTCAT-3′), GAPDH forward (5′-GACTCATGACCACAGTCCATGC-3′), and GAPDH reverse (5′-AGAGGCAGGGATGATGTTCTG-3′). The expression levels of SNHG15, miR-346, and TRAF4 were calculated by the 2^−ΔΔCt^ method, and glyceraldehyde-3-phosphate dehydrogenase (GAPDH) or U6 snRNA was viewed as an internal control for SNHG15, TRAF4, and miR-346.

#### 2.3.1 Subcellular fractionation assay

In this assay, Nuclear/Cytosol Isolation Kit (BioVision, San Francisco, CA, USA) was applied to isolate nuclear and cytoplasm fractions in line with the supplier’s instructions. RNAs isolated from the nucleus and the cytoplasm were analyzed by the qRT-PCR assay. The expression levels of circNHSL1, U6 (nucleus control), and GAPDH (cytoplasm control) were examined separately.

### 2.4 3-(4,5-Dimethyl-2-thiazolyl)-2,5-diphenyl-2-*H*-tetrazolium bromide (MTT) assay

The proliferation capacity of transfected SaoS2 and HOS cells was evaluated by the MTT assay. Briefly, transfected SaoS2 and HOS cells (2.5 × 10^3^/well) were seeded into 96-well plates (Corning Inc., Corning, NY, USA) and maintained in an incubator with 5% CO_2_ at 37°C for 24, 48, and 72 h. Then, MTT (20 µL) from Sigma (St Louis, MO, USA) was replenished to each well and kept for 4 h. Following this, the supernatant of each well was discarded, and DMSO (150 µL, Sigma) was added for the dissolution of formazan crystals. Finally the Microplate Absorbance Reader (Thermo Fisher Scientific, Waltham, MA, USA) was executed for the assessment of the optical density value at 490 nm.

### 2.5 Flow cytometry

Cells were collected by digesting with pancreatin and centrifuging and then resuspended with 1× binding buffer after being washed with iced phosphate-buffered saline (PBS), followed by supplementation with Annexin V-fluorescein isothiocyanate propidium iodide (Annexin V-FITC/PI) kit (BD Pharmingen, San Diego, CA, USA) following the manufacturer’s instructions. Apoptotic cells were examined using the flow cytometer (BD Biosciences, Franklin Lakes, NJ, USA), and then, the apoptosis rate was calculated.

### 2.6 Western blot

RIPA buffer (Solarbio, Beijing, China) was used to isolate total proteins in cells, and proteins were quantified by a NanoDrop 3000 (Thermo Fisher Scientific). Sodium dodecyl sulfate-polyacrylamide gel electrophoresis (SDS-PAGE) was used to separate proteins, and then, proteins were transferred onto polyvinylidene fluoride (PVDF) membranes. Membranes were then blocked using skim milk for 2 h at 37°C and then incubated with primary antibodies at 4°C overnight. Following 2 h incubation with the secondary antibody [Goat Anti-Rabbit IgG H&L (HRP) (1:1,000; ab205718, Abcam, Cambridge, UK)], the chemiluminescence was performed using an ECL detection kit (Beyotime). The primary antibodies were as follows: anti-Bcl-2 (1:1,000; ab196495, Abcam), anti-Bax (1:1,000; ab53154, Abcam), anti-Cleaved Caspase 3 (1:1,000; ab49822, Abcam), anti-TRAF4 (1:1,000; ab245666, Abcam), and anti-GAPDH (1:5,000; ab37168, Abcam).

### 2.7 Transwell assay

The rate of cell invasion was investigated by Transwell chamber (Corning) with Matrigel Matrix (Corning). The lower chamber was filled with RPMI-1640 medium with 10% FBS, while the transfected SaoS2 and HOS cells were injected into the upper chamber with 100 µL of serum-free medium, according to the manufacturer’s instructions. Finally, paraformaldehyde (PFA; Sigma) was used to attach cells located on the lower surface of the upper chamber. Cells were analyzed under a microscope after staining with crystal violet.

### 2.8 Dual-luciferase assay

The starBase or TargetScan database was executed for the prediction of the binding sites between miR-346 and SNHG15 or TRAF4, individually. Following this, the pGL3-control vector (Promega, Madison, WI, USA) with the wild-type SNHG15 sequence (SNHG15 WT) and mutant SNHG15 sequence (SNHG15 MUT) (with predicted miR-346 binding sites or not) was constructed to verify the binding sites between miR-346 and SNHG15. The miR-control or miR-346 was then co-transfected into OS cells with dual-luciferase reporter vectors for the execution of the dual-luciferase reporter assay. Similar steps were used for the identification of binding sites between miR-346 and TRAF4. Finally, the luciferase activities of luciferase reporter vectors were tested through the dual-luciferase reporter assay kit (Promega). The Renilla luciferase reporter vector was used as the internal reference.

### 2.9 Xenograft tumor model

A total of six BALB/c male nude mice, purchased from Charles River Laboratories (Beijing, China), were randomly divided into two groups (*n* = 3 per group). A total of 2 × 10^6^ HOS cells stably transfected with sh-SNHG15 were injected subcutaneously into the left hindquarter of the nude mice; the control group received an equal amount of sh-control. The tumor volume was measured using a caliper every 10 days. After 45 days, the mice were euthanized under anesthesia to separate the tumors for weighing, qRT-PCR, and western blot assay.


**Ethical approval:** The research related to animal use has been complied with all the relevant national regulations and institutional policies for the care and use of animals, and has been approved by the Experimental Animal Ethics Committee of Yijishan Hospital (The First Affiliated Hospital of Wannan Medical College).

### 2.10 Statistical analysis

All data were expressed as mean ± standard deviation (SD) and analyzed using SPSS 17.0 software. Comparisons among different groups were analyzed using paired Student’s *t*-test and one-way analysis of variance. A *P* value less than 0.05 was regarded as statistically significant.

## 3 Results

### 3.1 The role of lncRNA SNHG15 in OS tissues and cells

**Figure 1:**
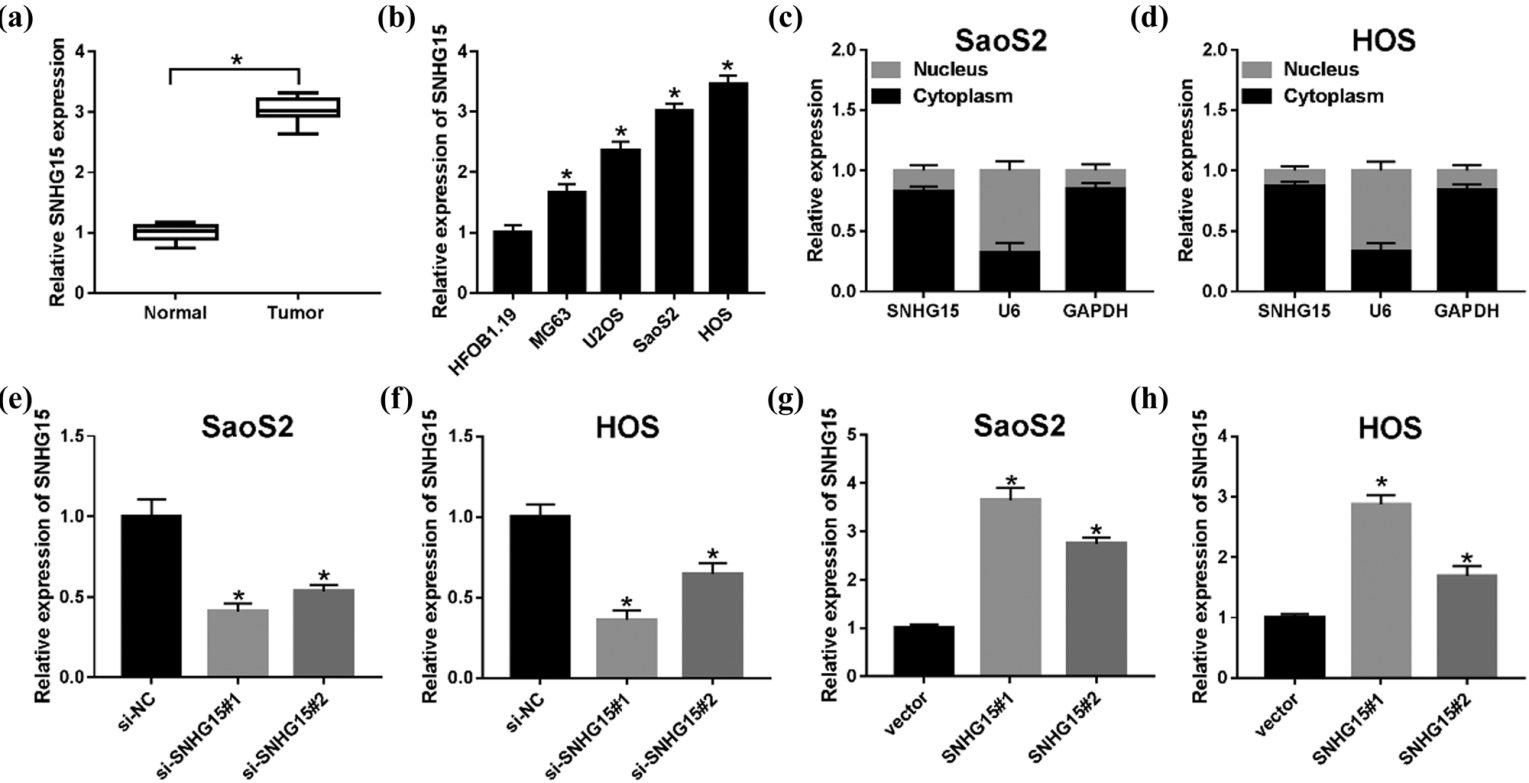
The roles of lncRNA SNHG15 in OS tissues and cells. (a) qRT-PCR was used to detect the expression of lncRNA SNHG15 in tissues from 30 patients with OS compared with the adjacent parts. (b) The expression of SNHG15 in human osteoblast cells (HFOB1.19) and OS cells (MG63, U2OS, SaoS2, and HOS) was measured by the qRT-PCR assay. (c and d) Localization of SNHG15 in SaoS2 and HOS cells was analyzed by the Subcellular fractionation assay. (e and f) The relative expression level of SNHG15 was detected in SaoS2 and HOS cells transfected with si-NC, si-SNHG15#1, and si-SNHG15#2. (g and h) The SNHG15 level was measured in SaoS2 and HOS cells transfected with vector, SNHG15#1, and SNHG15#2. *P < 0.05.

We evaluated the expression level of SNHG15 in OS tissues, and the results revealed that the expression of SNHG15 was significantly upregulated in OS tissues (Figure 1a). We also explored the expression pattern of SNHG15 in four different OS cell lines (MG63, U2OS, SaoS2, and HOS). The similar enhanced expression of SNHG15 could be seen in these cells (Figure 1b). Hence, the SaoS2 and HOS cells were chosen for the next experiments, owing to the ectopic expression of SNHG15 in SaoS2 and HOS cells versus HFOB1.19 cells. Moreover, the subcellular fractionation assay suggested enrichment of SNHG15 mainly in the cytoplasm (Figures 1c and d), implying SNHG15 can exert posttranscriptional regulation. Apart from that, qRT-PCR results indicated that SNHG15 level was decreased in SaoS2 (si-SNHG15#1: 41.2%, si-SNHG15#2: 53.6%) and HOS cells (si-SNHG15#1: 36.2%, si-SNHG15#2: 64.7%) (Figure 1e and f), suggesting that the knockdown efficiency of SNHG15 was successful in OS cells. Meanwhile, the transfection efficiency of SNHG15#1 and si-SNHG15#2 was also measured and presented in Figure 1g and h. These data indicated that SNHG15 might be involved with the progression of OS.

### 3.2 Knockdown of SNHG15 repressed proliferation and invasion, but induced apoptosis, in OS cells

**Figure 2:**
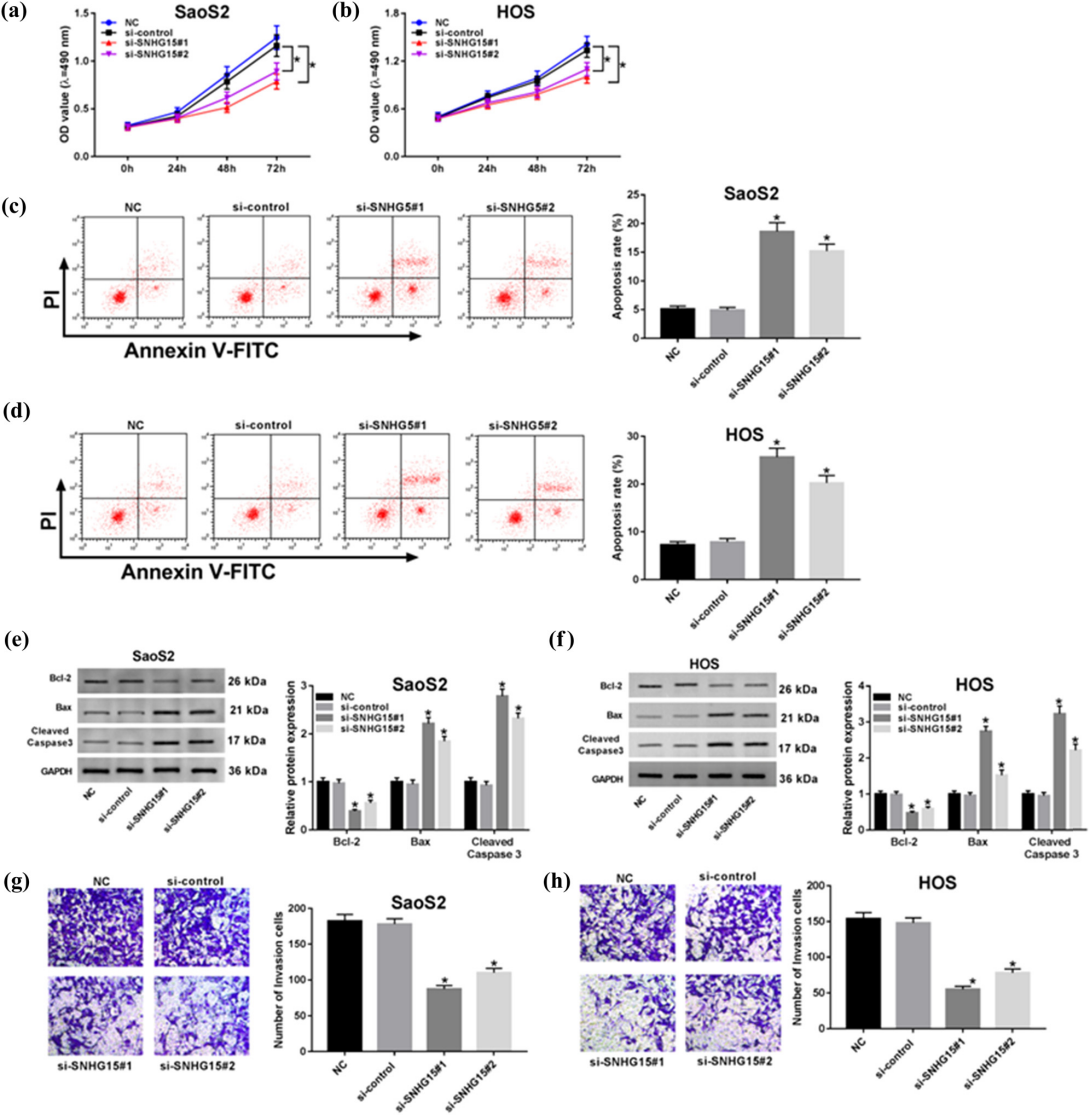
Knockdown of SNHG15 repressed the abilities of proliferation and invasion but induced apoptosis in OS cells. The OS cells were transfected with si-control or si-SNHG15. By the loss-function experiments, the biofunction effects of SNHG15 were performed. (a and b) The cell viability at determined times (24, 48 and 72 h) was analyzed by the MTT assay in OS cells. (c and d) The rate of apoptosis was measured by the flow cytometry assay. (e and f) The levels of apoptosis-related protein (Bcl-2, Bax, and Cleaved Caspase 3) were confirmed by western blot. (g and h) The cell invasion was evaluated by the transwell assay in SaoS2 and HOS cells. *P < 0.05.

First, to explore the role of SNHG15 of OS, we detected the biofunctional effects (proliferation, apoptosis, and invasion) (Figure 2) when cells were transfected with si-SNHG15 vector, and the results showed that proliferation and invasion were limited (Figure 2a, b, g and h) with apoptosis being promoted (Figure 2c and d). Second, we also investigated the change of apoptosis-related proteins in OS cells. The western blot assay confirmed that the levels of both Bax and Cleaved Caspase 3 were increased, while that of Bcl-2 was suppressed (Figure 2e and f). These data suggest that the knockdown of SNHG15 ameliorates the proliferation, apoptosis, and invasion abilities of OS cells.

### 3.3 MiR-346 was a target for SNHG15

**Figure 3:**
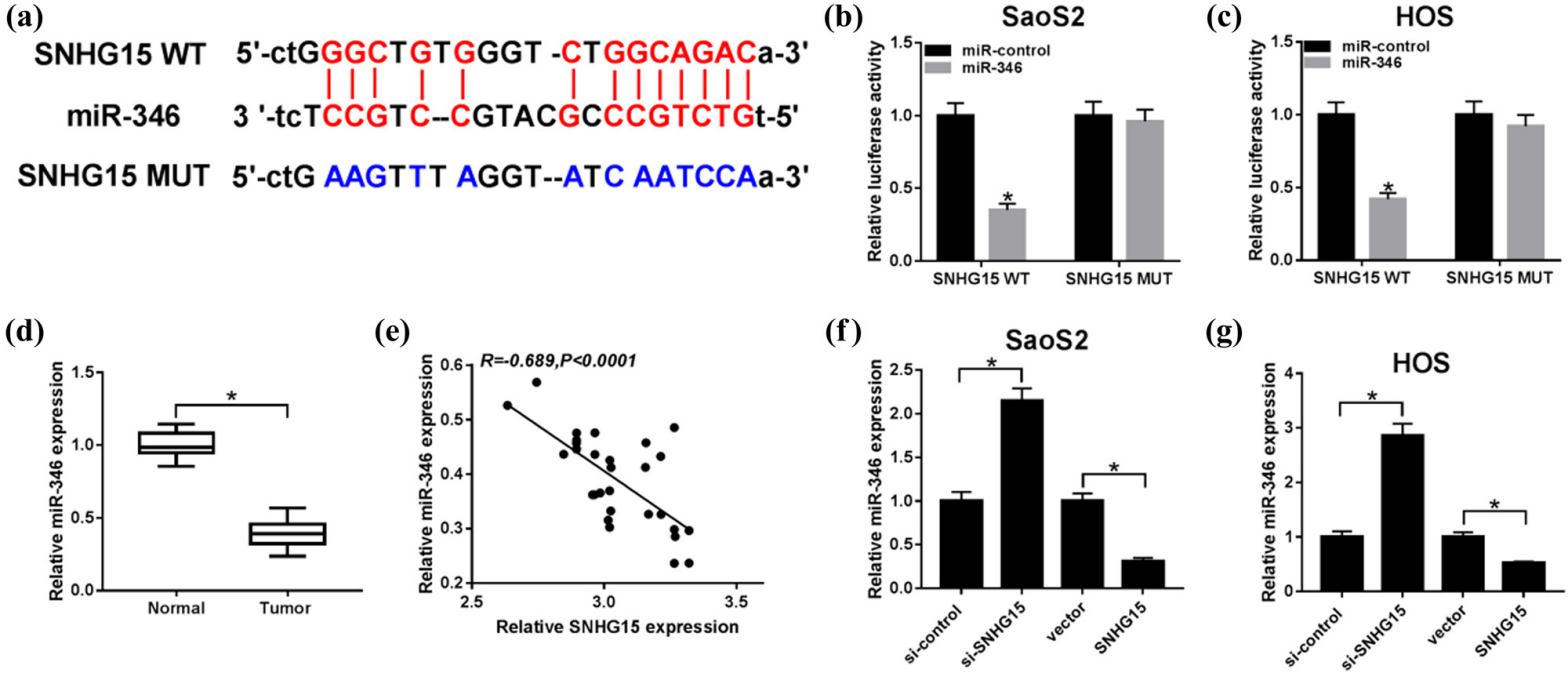
miR-346 was a target for SNHG15. (a) The putative binding sites between miR-346 and SNHG15 were predicted by starBase. (b and c) The predicted sites were identified by the dual-luciferase reporter assay. (d) QRT-PCR was carried out to detect the level of miR-346 in OS tissues. (e) A correlation between the expression of miR-346 and SNHG16 was explored. The SaoS2 and HOS cells were transfected with SNHG15 or vector. (f and g) The expression of miR-346 was detected by qRT-PCR in SaoS2 and HOS cells. *P < 0.05.

StarBase v 2.0 software was used to explore the potential binding sites of SNHG15 to miR-346 (Figure 3a). A dual-luciferase activity assay was performed in SaoS2 and HOS cell and showed that co-transfection of miR-346 and SNHG15 wide type (WT) significantly decreased the luciferase activity, whereas co-transfection of miR-control and SNHG15-mutant (MUT) did not change the dual-luciferase activity (Figure 3b and c). Meanwhile, we measured the expression pattern in OS tissues and interaction between miR-346 and SNHG15 and found an obvious decline in miR-346 in the tumor group compared with normal parts (Figure 3d), and a negative correlation could be seen between the expression of miR-346 and SNHG15 (Figure 3e). Then, the qRT-PCR assay was conducted to assess the expression of miR-346 after loss- and gain-function administration. The data suggested that the expression of miR-346 represented high tendency when cells transfected with si-SNHG15 vectors, while the low trend of miR-346 occurred when cells transfected with SNHG15 overexpressed vectors, compared with its own negative controls individually (Figure 3f and g).

### 3.4 Inhibition of miR-346 reversed the effects of downregulation of SNHG15 of OS *in vitro*

**Figure 4:**
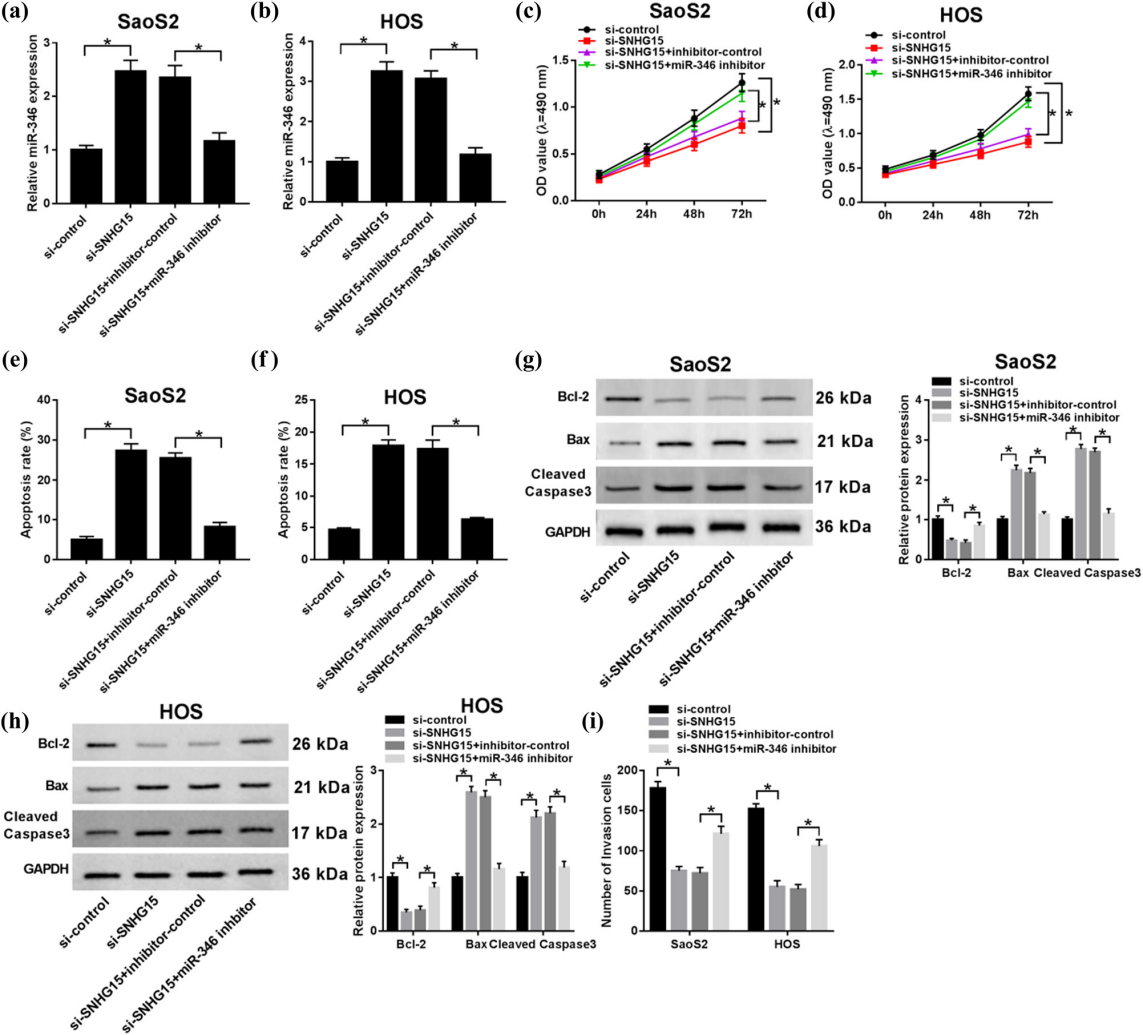
Inhibition of miR-346 inverted the effects from the downregulation of SNHG15 of OS *in vitro.* Si-control, si-SNHG15, si-SNHG15 + inhibitor-control, and si-SNHG15 + miR-346 inhibitor were separately transfected into OS cells. (a and b) The knockdown efficiency of miR-346 was confirmed by qRT-PCR. (c and d) The cell viability was analyzed by the MTT assay at stated times (24, 48, and 72 h) in SaoS2 and HOS cells. (e and f) Flow cytometry assay was used to confirm the rate of apoptosis. (g and h) Apoptosis-related proteins (Bcl-2, Bax, and Cleaved Caspase 3) were evaluated using western blot. (i) The cell invasion was evaluated by the transwell assay. *P < 0.05.

**Figure A1:**
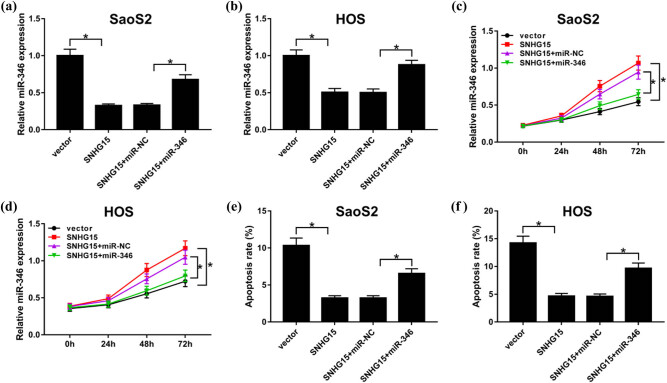
Overexpression of miR-346 alleviated SNHG15 upregulation-mediated cell viability and apoptosis in OS cells. (a and b) MiR-346 level was detected in SaoS2 and HOS cells transfected with vector, SNHG15, SNHG15+miR-NC, and SNHG15+miR-346. (c and d) Cell viability was assessed in transfected SaoS2 and HOS cells. (e and f) Apoptosis was examined in transfected SaoS2 and HOS cells.*P < 0.05.

To figure out how SHNG15 regulates the changes of miR-346 upon biofunctional phenotype, anti-miR-346 plasmid (miR-346 inhibitor) was constructed and transfected into the SaoS2 and HOS cells with downregulated expression of SNHG15, and the qRT-PCR assay confirmed that miR-346 inhibitor successfully suppressed the level of miR-346 in SaoS2 and HOS cells, compared with the transfection with an inhibitor-control (Figure 4a and b). It was observed that the suppression of miR-346 reversed the limitation of proliferation (Figure 4c and d) and invasion (Figure 4i), and the promotion of apoptosis had occurred in the cells where SNHG15 was downregulated (Figure 4e and f). When it referred to the influence on protein biomarkers of apoptosis, western blot results suggested that decreasing miR-346 inverted the enhanced expression pattern on Bax and Cleaved Caspase 3 and limited pattern on Bcl-2 from downregulation of SNHG15 in cells (Figure 4g and h). In addition, our data suggest that upregulating miR-346 could reduce the upregulating effect of SNHG15 on OS cell viability and the decrease in apoptosis rate in SaoS2 and HOS cells (Figure A1). These results show that limitation of miR-346 reversed the effects from downregulation of SNHG15 in OS cells.

### 3.5 TRAF4 was a direct target of miR-346

**Figure 5:**
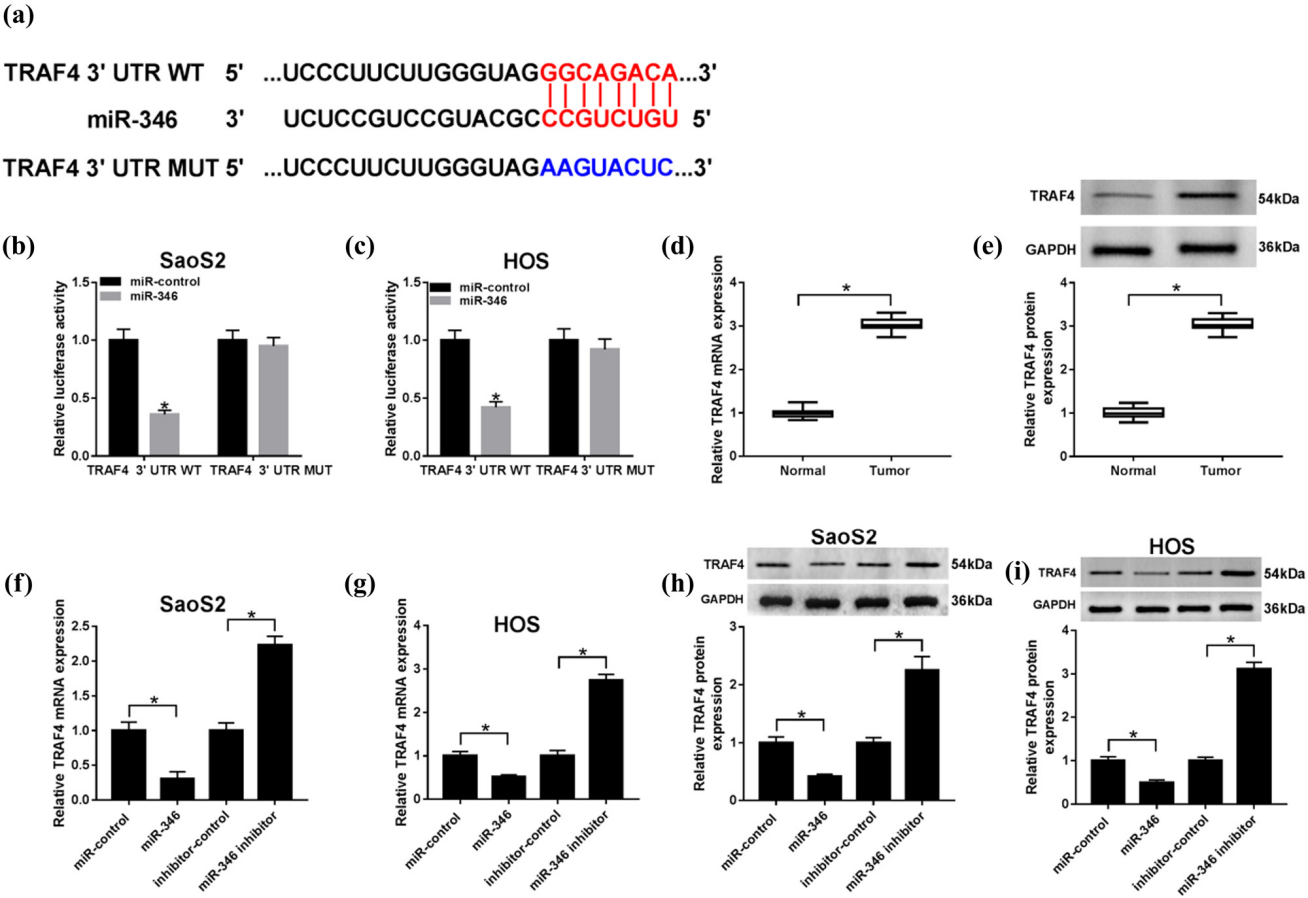
TRAF4 was a direct target of miR-346. (a) TRAF4 was predicted by starBase as a potential target for miR-346. (b and c) The dual-luciferase reporter assay was conducted to verify the interaction between miR-346 and TRAF4. (d and e) The expression of TRAF4 on OS tissues was explored at mRNA (d) and protein (e) levels. (f–i) The expression of TRAF4 after the loss-and-gain experiment was measured in OS cells using qRT-PCR (f and g) and western blot (h and i). *P < 0.05.

To further figure out the role of miR-346 upon regulatory mechanisms in OS, the online predicted bioinformatics software starBase (http://starbase.sysu.edu.cn/starbase2/) was used to predict the potential gene-binding sites in miR-346. We speculated that TRAF4 had a binding site with miR-346 (Figure 5a). To confirm this relationship between TRAF4 and miR-346, the dual-luciferase reporter assay showed that co-transfection of miR-346 and TRAF4-WT decreased the luciferase activity, while co-transfection of miR-control and TRAF4-MUT did not change the dual-luciferase activity (Figure 5b and c). Interestingly, the expression of TRAF4 on mRNA (Figure 5d) and protein (Figure 5e) levels was elevated in OS tissues as well. Then, we explored the expression of TRAF4 in SaoS2 and HOS cells, by gain-functional experiments, and found that the level of TRAF4 was upregulated with the limited expression of miR-346 (including mRNA (Figure 5f and g) and protein levels (Figure 5h and i)) when the cells were successfully transfected with the miR-346 inhibitor. These data confirmed that TRAF4 is a target of miR-346, and miR-346 regulates the expression of TRAF4 in a negative manner.

### 3.6 Overexpression of TRAF4 restores the biofunctional results from upregulation of miR-346

**Figure 6:**
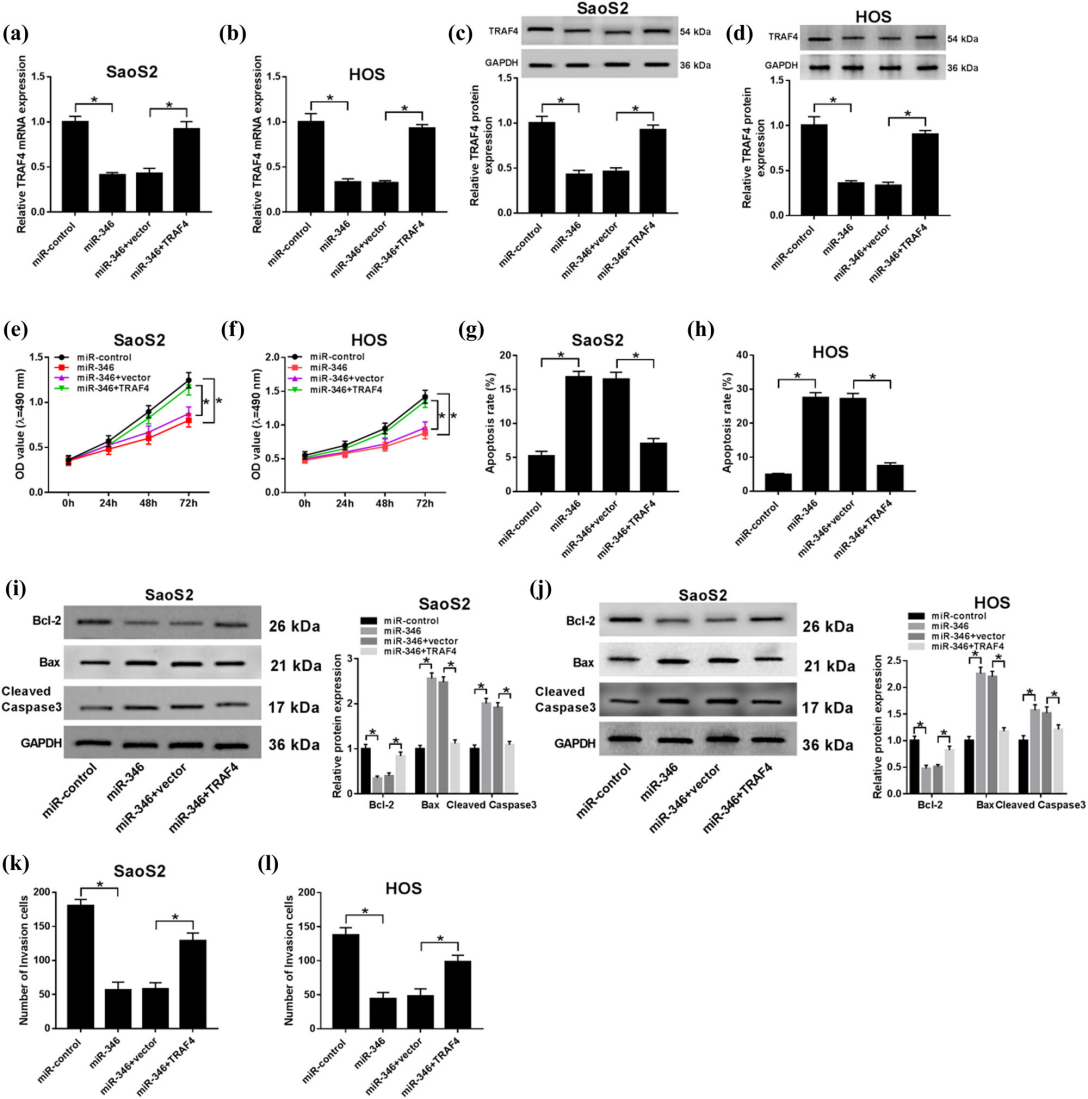
Overexpression of TRAF4 restored the biofunctional results from the upregulation of miR-346. (a–d) The SaoS2 and HOS cells were administrated by co-overexpression miR-346 and TRAF4, and the TRAF4 expression was measured by qRT-PCR (a and b) and western blot (c and d). (e–l) The functional assays, namely, proliferation (e and f), apoptosis (g and h), and invasion (k and l) were determined by MTT analysis, flow cytometry assays, and transwell invasion assay individually. (i and j) The biomarkers of Bcl-2, Bax, and Cleaved Caspase 3 were assessed by the western blot. *P < 0.05.

To confirm that the expression of TRAF4 could be regulated by miR-346, we examined the mRNA level of TRAF4 by the gain-functional experiment. As shown in Figure 6a and b, the mRNA level of TRAF4 was increased when cells were co-transfected with miR-346 and TRAF4, compared with cells co-transfected with miR-346 and vector, which was assayed by the qRT-PCR. Then, the equal results were shown in Figure 6c and d at the protein level. The function assays also affirmed that the inhibition of proliferation (Figure 6e and f) and invasion (Figure 6k and l) by miR-346 was impaired by overexpression of TRAF4 in OS cells. Moreover, the promotion effect of miR-346 overexpression on cell apoptosis was abolished by upregulation of TRAF4 *in vitro* (Figure 6g and h). The high expression of Bax and Cleaved Caspase 3 and low expression of Bcl-2 were inverted by the enforced expression of TRAF4 in OS cells (Figure 6i and j). The results suggested that overexpression of TRAF4 restored the biofunctional results from upregulation of miR-346.

### 3.7 SNHG15 regulates the expression of TRAF4 by sponging with miR-346

**Figure 7:**
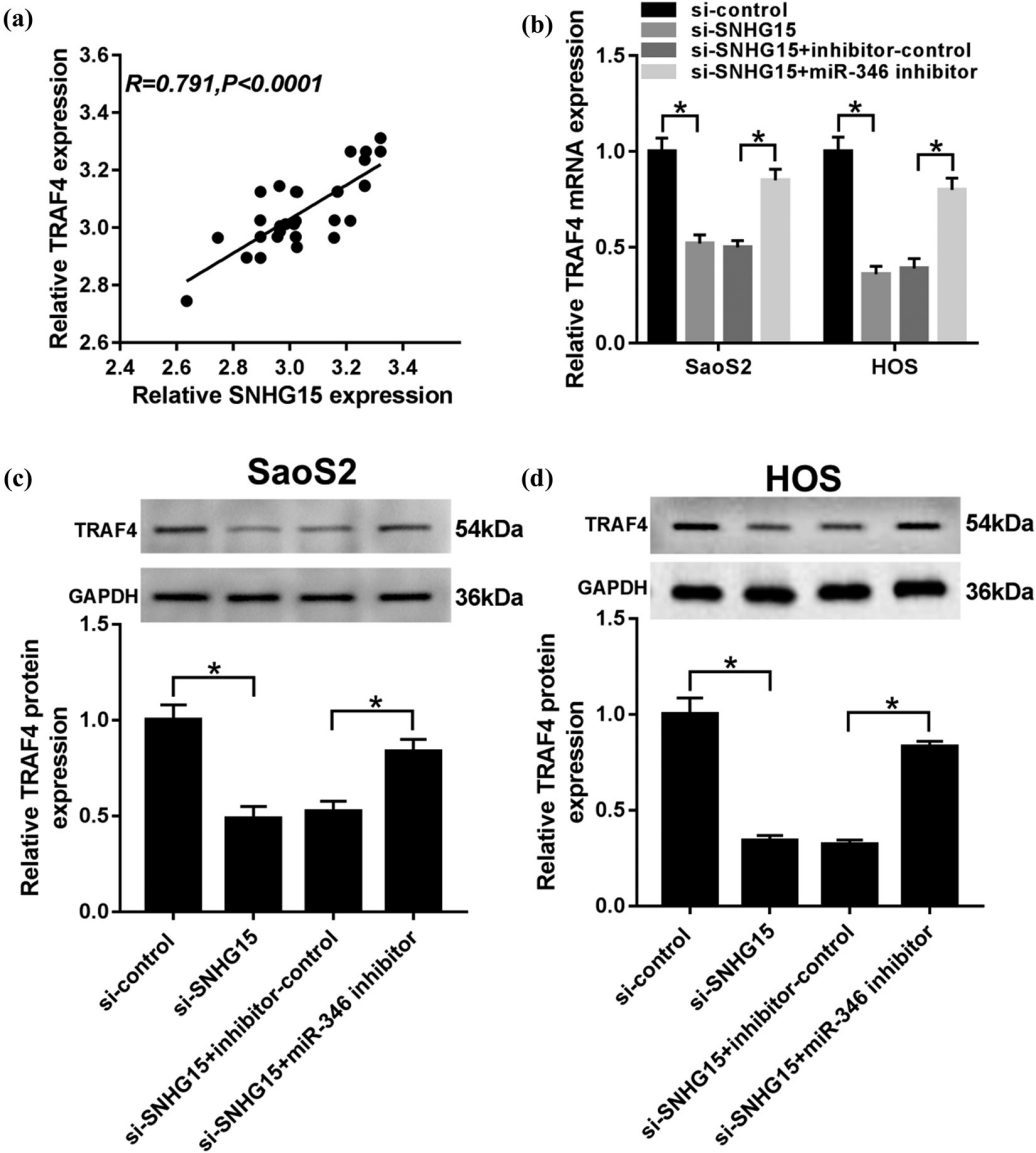
SNHG15 regulated the expression of TRAF4 by sponging with miR-346. (a) The relationship between TRAF4 and SNHG15 was uncovered using the qRT-PCR analysis. (b–d) The SaoS2 and HOS cells were co-transfected with si-SNHG15and miR-346 inhibitor, and the expression pattern of TRAF4 was investigated by the qRT-PCR (b) and western blot separately (c and d). *P < 0.05.

To investigate how SNHG15 regulates OS progression via the TRAF4/miR-346 axis, we thus designed the next experiments. We found that the relative expression of TRAF4 and SNHG15 maintain a positive correlation (Figure 7a). The mRNA level and protein level of TRAF4 were decreased in OS cells treated with si-SNHG15, while miR-346 inhibitor could mitigate the suppression effect (Figure 7b–d). These data identified that SNHG15 could regulate the expression of TRAF4 by targeting miR-346 in OS cells.

### 3.8 Silencing of SNHG15 inhibits OS growth *in vivo*

**Figure 8:**
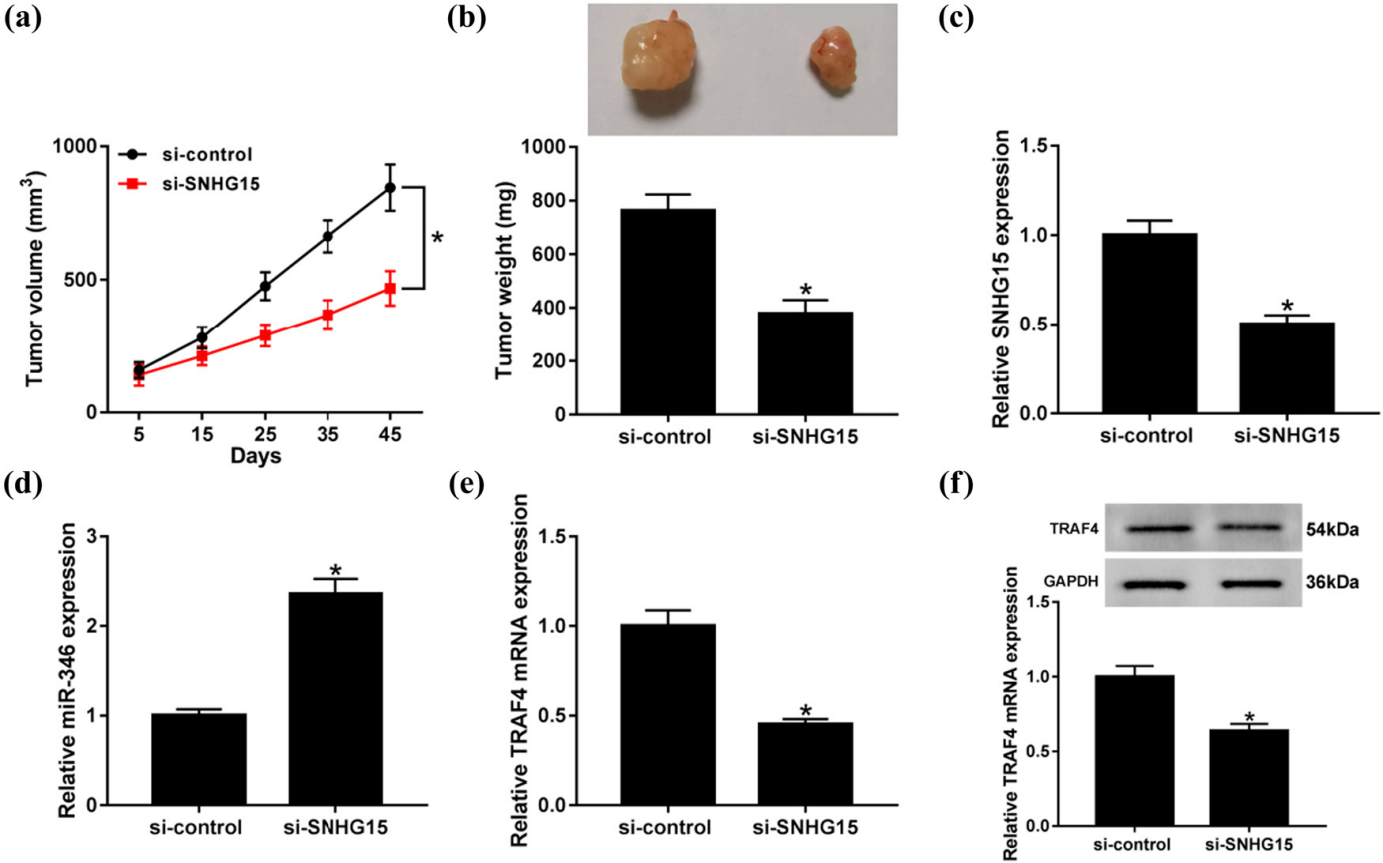
Silencing of SNHG15 inhibited the growth of OS *in vivo*. Mice (3 mice/group) were subcutaneously injected with HOS cells (2 × 10^6^) stably transfected with sh-SNHG15 or an equal volume of the vehicle into the left flank; the animals were sacrificed after 45 days. (a and b) Tumor volume (a) and tumor weight (b) were measured. (c–e) The qRT-PCR analysis was performed to measure the expression levels of SNHG15 (c), miR-346 (d), and TRAF4 (e) in excised tumor tissues. (f) Western blotting was performed to measure the expression of TRAF4. *P < 0.05.

To further examine the efficacy of SNHG15 *in vivo*, as shown in Figure 8a, the tumor in sh-SNHG15-transfected group grew more slowly compared to that in the sh-control group. A significant decrease in the tumor weight was observed between sh-control and sh-SNHG15 groups (Figure 8b). Excised tumor tissues exhibited the improved expression of miR-346 and the reduced expression of SNHG15 in the sh-SNHG15 group (Figure 8c and d). In addition, downregulation of SNHG15 significantly inhibited the expression of TRAF4 at mRNA and protein levels (Figure 8e and f). These results suggested that silencing of SNHG15 inhibits the OS growth *in vivo*.

## 4 Discussion

LncRNA SNHG15 has been reported to be a cancer-promoting lncRNA in a variety of tumors [25], for instance, prostate cancer [26], colorectal cancer [27], ovarian cancer [28], lung cancer [29,30], and renal cell carcinoma [19]. Zhang et al. identified that SNHG15 was elevated and promoted cell proliferation in prostate cancer cells [26]. Jin et al. reported that SNHG15 promotes proliferation, apoptosis, cell cycle, and tumor growth in non-small cell lung cancer *in vitro* and *in vivo* [30].

In this experiment, we first examined the expression level of lncRNA SNHG15 in OS cells and tissues, and the results showed that the expression of lncRNA SNHG15 is upregulated in both tissues and cells, consistent with the previous reports [14].

In previous studies, downregulation of SNHG15 was shown to inhibit tumor cell proliferation and invasion and promote apoptosis [14,31]. Through loss-functional experiments, similar results were found in our experiments that the downregulated expression of SNHG15 slowed down the abilities of proliferation and invasion in OS cells and promoted the rate of cell apoptosis. Subsequently, the miR-346 was predicted and proved to be the potential target for SNHG15. Interestingly, the expression pattern of miR-346 was attenuated in OS tissues and negatively correlated with that of SNHG15. Hence, we conducted further exploration by constructing inhibitor plasmid and uncovered that the knockdown of miR-346 could reverse the effect of decreasing SNHG15.

MiRNAs are a variety of endogenous low-molecular-weight compound, with about 22 nucleotides in length. Evidence is increasingly supporting that some miRNAs exert a tumor-suppressing role through targeting and inhibiting the expression of multiple oncogenes. The oncogene TRAF4 has been widely studied in a variety of cancers, such as hepatocellular carcinoma [32] cholangiocarcinoma [33], and non-small cell lung cancer [34]. Kang et al. showed that TRAF4 was upregulated in cholangiocarcinoma tissues and positively correlated with tumor differentiation and TNM (Tumor, Lymph Node, Metastasis) stage [33]. Yang et al. showed that the level of TRAF4 mRNA was inversely correlated with miR-302c-3p expression in hepatocellular carcinoma specimens, and TRAF4 restoration reversed the inhibitory effect of miR-302c-3p on AKT-induced EMT and hepatocellular carcinoma cell metastasis [32]. It has been reported that the upregulated TRAF4 was present in cholangiocarcinoma [33]. Consistent with the previous reports, the expression level of TRAF4 was promoted in OS tissues at both mRNA and protein levels in our study. By targeted prediction and biological verification, we found that overexpression of TRAF4 reversed the effects of miRNA-346 on OS cells. *In vivo* experiments also showed that the lncRNA SNHG15 knockdown promotes the OS tumor growth through sponging miR-346 to allow TRAF4 expression.

There were some limitations in this study. First, the interaction between miR-346 and SNHG15 or TRAF4 was initially detected by the dual-luciferase reporter assay, and it should be confirmed by RNA immunoprecipitation or RNA pull-down. Besides, the results and conclusions obtained using commercial cell lines could not fully represent the actual situation *in vivo*.

In summary, we identified that the highly expressed cancer-promoting lncRNA SNHG15 plays an essential role in the development of OS. Furthermore, our study first highlighted the role of lncRNA SNHG15 in promoting TRAF4 by sponging with miR-346, thereby promoting the progression of OS. Thus, this molecule is a new therapeutic target for OS.
